# Advances in Prevention, Screening, and Early Detection of HPV-Associated Head and Neck Cancers

**DOI:** 10.3390/v17101339

**Published:** 2025-10-05

**Authors:** Evangelos Zazas, Panagiota Economopoulou, Ioannis Kotsantis, Anastasios Kyriazoglou, Menelaos G. Samaras, Periklis Foukas, Amanda Psyrri

**Affiliations:** 1 Oncology Department, 2nd Propaedeutic Clinic of Internal Medicine, Attikon University Hospital, National and Kapodistrian University of Athens, Rimini 1, Chaidari, 124 62 Athens, Greece; 2 Second Department of Pathology, Attikon University Hospital, National and Kapodistrian University of Athens, Rimini 1, Chaidari, 124 62 Athens, Greece; mgsamaras@med.uoa.gr (M.G.S.);

**Keywords:** HPV, head and neck cancer, oropharyngeal carcinoma, screening biomarkers, liquid biopsy, HPV serology, circulating tumor DNA, salivaomics, early detection, prevention

## Abstract

HPV-related head and neck cancers are increasing globally and although they constitute a major public health problem, there are currently no validated screening or early detection methods in widespread clinical use. This review discusses advances in clinical and molecular aspects of prevention, screening, and early detection of HPV-related head and neck cancers (HNCs), such as potential use of HPV blood or saliva seropositivity, RNA biomarkers, liquid biopsy, circulating tumor DNA, and proteomics. In addition to HPV vaccination, public education about vaccination, smoking, and safe sexual practices is warranted. Continued research is warranted to define optimal use and integration of approaches for prevention, screening, and early detection methods of HNCs.

## 1. Introduction

HPVs are associated with 5% of cancers worldwide [1,2], such as anogenital and oropharyngeal [3]. HPV-related head and neck carcinomas differ from HPV-negative disease, and there are opportunities for non-invasive early screening [4], with serum biomarkers such as p16 overexpression, HPV E6/E7 serology, and circulating HPV DNA [5]. In the United States and other developed countries [6], HPV is detected in over 60% of oropharyngeal squamous cell carcinomas (OPSCC) [7]. Early screening leads to better survival [8]. In this review we discuss the most recent developments in epidemiology, risk factors, oncogenicity, prevention, screening, and early detection methods of HPV-positive HNSCC [9].

### Methods

This narrative review was conducted through a structured search of PubMed and Scopus databases to identify original research, reviews, and case reports in English publications through July 2025. The following keyword phrases were used: human papillomavirus, HPV, head and neck cancer, oropharyngeal cancer, screening, biomarker, early detection, liquid biopsy, circulating tumor DNA, RNA biomarkers, HPV-vaccination, and HPV-serology. Reference lists of selected articles were also screened to identify additional relevant studies. Priority was given to recent studies and large cohort or multicenter investigations, and publications in peer-reviewed journals.

## 2. General Risk Factors for HPV-Related Head and Neck Carcinomas

General risk factors for HPV-related HNCs include tobacco and alcohol usage [10], male sex [6,11], poor dietary habits [12], nutritional deficits [13], older age [14], lower socioeconomic status [15], specific geographic regions [10], co-infections, chronic immunodeficiencies, and high risk sexual behaviors [16,17], but the key determinant is infection with high-risk HPV types [18]. Most oral HPV infections are transient and clear spontaneously; however, viral persistence is the critical factor that drives malignant transformation [19]. 

## 3. Oncogenic Function of HPV

HPV is a DNA virus with tropism [20] for squamous epithelia [21]. HPV-16 and HPV-18 are associated with premalignant squamous intraepithelial neoplasia that may progress to malignancy [21]. Decreased E2 protein leads to higher expression of viral oncoproteins E6 and E7 (which inactivate tumor suppressor genes pRb and p53). E7 binding to pRb releases transcription factor E2F from the pRb-E2F protein complex, promoting cell cycle progression and the release of p16INK4A. Hence, high p16INK4A expression is a reliable surrogate marker for high-risk HPV infection [22]. HPV E6 and E7 oncoproteins drive oncogenesis by preventing cell death, sustaining proliferating signaling, and evading apoptosis and growth suppressor activity. E6 degrades p53, while E7 inhibits pRb protein. HPV may persist in two forms: integrated into the host genome (~60%) or episomal (~40%) [23]. Integration of HPV DNA into the host genome is associated with genomic instability and may influence prognosis, with fully integrated HPV linked to poorer outcomes in some cohorts [23,24].

## 4. Primary Prevention

### HPV Vaccination as a Method of Primary Prevention and Current Vaccination Rates

Vaccines targeting HPVs induce antibody-mediated immunity against HPV capsid antigens [25]. Gardasil-VR, Gardasil-9VR, and Cervarix-VR are approved by the US Food and Drug Administration (FDA) for cervical cancer prevention and target the most common HPV types associated [26]. They may also protect against HPV-related HNC, though the latency period to malignancy may be 10–30 years [27]. A randomized trial in Costa Rica showed that Cervarix was effective in preventing HPV-16 and 18 oral infections [28]. 

The US CDC compared HPV infection rates before vaccination (2003–2006) and after vaccination (2009–2012) [29]. The prevalence of HPV-6, 11, 16, and 18 decreased by 64% in sexually active girls and women aged 14–19 years and by 34% in those aged 20–24 years. Vaccination rates, particularly among men, are below the desired level in many countries [28]. In 2020 the FDA approved the use of Gardasil9 vaccine in the US for preventing HPV-associated oropharyngeal and HNSCCs; ongoing randomized trials are investigating its efficacy [26]. Macilwraith et al. reported that gender-neutral HPV-vaccination reduced the incidence of oropharyngeal cancers [30]. The full impact may be delayed given the latency. Nielsen et al. similarly reported that vaccination led to a reduction in oral and oropharyngeal HPV infections [31]. A considerable proportion of participants in case–control studies developed IgG antibodies in their oral cavity after receiving HPV vaccinations [31]. This may be a considerable tool to assess the efficacy of vaccination. DeKloe et al. examined a large patient population from the TriNetX US Collaborative network and showed early evidence that vaccination prevents development of several types of cancers including head and neck carcinomas [27]. There are disparities among countries and regions. Adekanmbi et al. showed a geographic disparity in vaccination rates across Texas [32]. Suboptimal vaccination rates remain a barrier for primary prevention in many countries, and the goal for more universal vaccination has not been met.

## 5. Screening Approaches

There are barriers to implementation of large-scale screening programs [33]^,^ including the fact that a person being HPV+ does not mean he or she will develop HPV-related malignancy. There is still no consensus on the ideal population to screen (e.g., all >45 years old vs concentrating on higher risk groups) [33,34]. HPV positivity does not equate to HNC, as only a subset of infections progress to malignancy [35]. HPV infection is common in the general population, and most infections are transient and asymptomatic. There have been discussions but not agreement on the suitable cost-effective screening programs [34]. We will discuss conventional and novel screening approaches and many biomarkers that could be used.

### 5.1. Conventional and Traditional Screening Methods

Today, a clinically suspicious lesion may be assessed for HPV status via p16 immunohistochemistry, HPV-DNA in situ hybridization, and HPV-PCR [36]. However, there are promising emerging tests utilizing PCR to analyze fine needle aspirates, saliva, brush cytology, and serum for HPV [37].

### 5.2. Commercially Available Kits

Several commercially available diagnostic kits are used for the detection of HPV in head and neck squamous cell carcinoma (HNSCC) [5]. The most widely adopted methods include:

- P16 Immunohistochemistry kits (e.g., CINtec^®^ Histology, Ventana BenchMark, Ventana Medical Systems, Tucson, AZ, USA);
- HPV DNA detection kits, like PCR-based kits (e.g., Roche Cobas^®^, Roche Diagnostics, Basel, Switzerland, HPV Test, Abbott RealTime High-Risk HPV, Abbott Molecular Inc, Abbott Park, IL, USA).
- In situ hybridization (ISH) kits (e.g., INFORM HPV III Family 16 Probe, Ventana Medical Systems, Tucson, AZ, USA);
- HPV E6/E7 mRNA detection kits, including RNAscope^®^ (Advanced Cell Diagnostics, Gateway Blvd, Newark, CA, USA)
- HPV HR, liquid-based assays, like the Aptima^®^ HPV Assay (Hologic Inc., Malborough, MA, USA);
- Droplet digital PCR (ddPCR) assays for ctHPVDNA [38,39,40].

### 5.3. Biomarkers for HPV Screening

We will review the many biomarkers used in the diagnosis, surveillance follow-up, and possible screening of HNCSCC [41,42]. Table 1 summarizes the most important ones.

#### 5.3.1. Seropositivity for HPV

Seropositivity is a reliable marker for HPV infection [43]. Strong correlations between prevalent HPV infection and seropositivity were reported in adults in many studies [44]. The proportion of participants with HPV seropositivity was significantly higher among cancer cases, particularly among those with OPSCC [45]. Seropositivity for HPV16 E6 has proven to be a reliable indicator for HPV-driven OPC [43]. Blood-based HPV16 E6 antibodies can be detected several years prior to diagnosis [46].

#### 5.3.2. Tumor-Derived HPV DNA in Saliva

A study of patients with OPC showed that tumor-derived viral DNA using the clinically validated ddPCR assay (NavDx) was detectable and quantifiable in 44 of 46 saliva samples (and also present in 43 of the plasma samples) [47,48]. Wasserman reported 29 patients positive for salivary HPV DNA, mostly high-risk subtypes, with 100% specificity and predictivity for p16+ OP tumors [49]. In a Florida study of 204 patents with OPC, oral gargle samples detected the HPV genotype in 83% of oral gargle samples and 93% of tumor specimens [50].

#### 5.3.3. RNA-Based Biomarkers

Different RNA types are explored as tools for both diagnosis and screening of HNSCCs [51]. These include mRNA (messenger RNA), miRNA (microRNA), snoRNA, (small nucleoral) lncRNA (long-non-coding) [52], snRNA (small nuclear), circRNA (circular RNA), piRNA (PIWI-interacting RNA), and others.

MicroRNAs are about 22 nucleotide non-coding RNAs that are involved in post-translational gene regulation [53] and hold a unique combination of properties including high specificity and presence in the bloodstream and a long lifetime [54]. Serum and salivary miRNA may distinguish between individuals with HPV and those who developed cancer [55]. Specific alterations in salivary miRNA expression may detect patients who have developed HPV-associated malignancies [56] by comparing the patterns of salivary miRNA expression between HPV-positive OPC patients and HPV-positive controls [57].

Small nuclear RNAs are a class of non-coding RNAs, typically 100–300 nucleotides in length, that are essential components of the spliceosome—the molecular machinery responsible for the splicing of pre-mRNA in eukaryotic cells [58]. There are few reports on the direct use of snRNA in HNSCC liquid biopsy, though there is potential for its application in HNSCC. Aberrant splicing events in cancer have been observed, resulting in the generation of isoforms that could potentially contribute to tumor progression [59]. Furthermore, snRNA’s involvement in alternative transcription, which when dysregulated may act as a carcinogenic factor in HNSCC, suggests the possible diagnostic value of these molecules [60].

Small nucleolar RNAs (snoRNAs) are a type of small RNA molecules that primarily reside in the nucleolus [61]. They play many roles in the chemical modification and processing of other RNA molecules, particularly ribosomal RNAs (rRNAs) and small nuclear RNAs. In HNSCC, down-regulated snoRNA expression levels have been found to significantly correlate with patient overall survival [62]. Research is performed to investigate the role of snoRNA in early diagnosis [63].

CircRNAs regulate proliferation by interfering with PI3K/Akt/mTOR [64], insulin-like growth factor, transforming growth factor (TGF) β, p53 signaling pathways, and apoptosis [65,66]. The circRNAs are dysregulated in patients’ body fluids compared to healthy controls. These changes were observed in both upregulated and downregulated circRNAs [67]. The expression levels of most circRNAs were found to be correlated with important clinical factors such as tumor size, tumor differentiation, distant metastasis, and stage [68].

PIWI-interacting RNAs (piRNAs) are a distinct class of small non-coding RNAs, typically 24–31 nucleotides in length, that bind to PIWI proteins and are best known for their role in silencing transposable elements and maintaining genomic stability, especially in germ cells [69,70]. The expression of specific piRNAs is found to be deregulated and varies with both the HPV status and type [71]. A five-piRNA signature has been identified, which can effectively distinguish a subset of HPV-positive HNSCC patients with poor outcomes.

#### 5.3.4. Circulating Tumor DNA

ctDNA-based assays may secure a molecular diagnosis at an earlier stage [72]. Siravegna reported in a study of 61 tumor patients that an institutionally generated ctDNA had a sensitivity of 98%, a specificity of 99%, a 98% positive predictive value, and a 99% negative predictive value in detecting HPV+ HNSCC [40] and proposed it to be both cost-effective and more accurate than FNA biopsy. The high sensitivity was seen even in T1N0 OPSCC. There are however limitations in its use [73].

#### 5.3.5. Exosomes

Exosomes play an important role in the tumor microenvironment and have been studied as potential biomarkers in HNSCC, as their concentration correlates with disease activity and tumor stage [65,66].

MicroRNAs in blood and saliva [74] are also concentrated in exosomes [75], and are implicated in cellular translational suppression, mRNA degradation, and transcriptional regulation. In a study conducted by Langevin et al., exosomes were isolated from patients with HNSCC and compared to those from noncancer patients and control cells. The study found that certain miRNAs, including miR-486-5p, miR-486-3p, and miR-10b-5p, were significantly elevated in exosomes secreted solely by cancer cells in culture [76].

Tumor cells derived from HNSCC may release substantial amounts of small extracellular vesicles (sEVs), particularly tumor-derived exosomes (TEX). These TEX possess immunosuppressive functions and facilitate immune evasion [65]. Exosomes differ from other extracellular vesicles in the blood, such as microvesicles or apoptotic bodies, in terms of their functional characteristics [77]. Previous studies have shown that specific sEV features, such as protein content and immunosuppression of T cells, are influenced by the disease activity of head and neck cancers [78].

#### 5.3.6. Proteomics

A proteomic-based approach has been used in different settings for the identification of the risk of treatment response, metastasis, and recurrence in HNSCCs [79] and for early detection. Models include special proteins, e.g., DHB12, HMGB3, and COBA1. Differences in the intensity levels of these proteins are found to be correlated with recurrence and the development of metastasis. Integrating proteomic predictive models into routine clinical practice could lead to more precise and individualized management of HNSCC [80]. Proteomics can also be used in the saliva with promising results [81]. Saliva contains specific hormones, antibodies, enzymes, and cytokines that are secreted by oral cancer cells or host cells. These proteins serve as potential targets for non-invasive screening [82].

#### 5.3.7. Salivaomics

Salivaomic approaches are discovery methods of potential biomarkers derived from salivary genomics/epigenomics, proteomics [83], transcriptomics, metabolomics, and microbiomics [84]. Some of the commercially available assays may predict the presence or absence of oral cavity cancer [85]. One proposed platform reported sensitivity of 90% and specificity of 62% measuring soluble CD44 and total protein content in oral rinses [86]. Another system examines six salivary messenger RNA (mRNA) markers (IL-1, IL-8, OAZ1, SAT1, S100P, and DUSP1) [87]. Other techniques using saliva, like RT-qPCR, have also shown promising results in the early detection and management of early-stage HPV-related cancer [88].

#### 5.3.8. HPV-Whole Genome Sequencing ctDNA Approach

Bryan et al. reported a 99% sensitivity and specificity of a multifeatured HPV whole-genome sequencing liquid biopsy (of 152 HPV+ HNSCC patients with controls) when compared with current screening methods for early cancer detection [89].

#### 5.3.9. Urine-Based Diagnostics

ctDNA in urine was tested as a noninvasive liquid biopsy for HPV+ oropharyngeal cancer, though several limitations like the ultrashort fragments were noted [90].

#### 5.3.10. Exhaled Breath Analysis

Exhaled breath analysis identifies volatile organic compounds in breath that are byproducts of cancer cells [91]. Several analytical methods, including gas chromatography and electronic nose sensor arrays, have been used to distinguish breath samples from cancer patients compared to controls [92].

## 6. Discussion

HNSCC incidence, morbidity, and mortality continues to rise [93]. HPV vaccination has reduced the incidence of cervical cancer, but in HPV-associated HNSCC, the impact is promising but less clear, and vaccination need to include boys as well as girls. National HPV vaccination programs have been implemented in 122 of 195 (63%) World Health Organization (WHO) member states as of 2022 [94], but fewer are vaccinated in low- and middle-income countries [95]. Early detection and screening are obviously important [96], but neither the optimal target population for HNSCC screening nor the best screening methods have been well-defined.

Serum biomarkers such as seropositive HPV+ [97] and techniques such as ctDNA [73] are promising. Newer diagnostic tools such as different types of RNAs differentially expressed are being evaluated as early detection tools. To overcome the limitations of a single biomarker, research is increasingly integration multiomics approaches. Artificial intelligence (AI) for data analysis will likely also be helpful.

The methods discussed in this review will likely see increasing implementation in HNSCC screening over time. Validating these tools will require well-designed research studies before acceptance into clinical practice. Prospective trials are needed to demonstrate efficacy and show screening improves patient outcomes in HPV-related head and neck cancer. It will be important clinically to define clear cost-effective guidelines that may differ depending on available resources [98]. 

## 7. Conclusions

HPV-associated HNSCC is a major and rising concern worldwide, and currently there are no well-established population-based screening methods—a gap that, if filled, could allow much earlier diagnosis. Newer molecular diagnostic tools like HPV serology, liquid biopsy, and other methods under development are promising tools in the early screening of high-risk populations. Continued research and well-designed trials will be crucial in translating these emerging technologies into effective screening programs.

## Figures and Tables

**Table 1:** Summary of the most important biomarkers for screening and early detection.

Biomarker	Sample Type	Validation Status	Pros	Cons
HPV16 serology	Blood	Validated (high for E6)	Detects early infection; non-invasive	Not all positives develop cancer
HPV DNA	Saliva	Partially validated	Non-invasive; site-specific	Limited sensitivity
Circulating tumor DNA (ctDNA)	Plasma	Clinical trials; validated in some studies	Detects low tumor burden; highly specific	Expensive; limited use
RNA biomarkers	Saliva/Blood	Experimental to partially validated	Non-invasive; potential specificity	Many remain unvalidated
Exosomes	Blood/Saliva	Early studies	Reflect tumor stage; non-invasive	Lack of standardization
Proteomics	Tissue/Saliva	Validated in some studies	High diagnostic accuracy	Requires specialized labs

## Data Availability

The original data presented in the study are openly available in PubMed and Scopus databases.
